# Association of Oxidative Stress Biomarkers with Gestational Diabetes Mellitus in Pregnant Women: A Case-Control Study

**DOI:** 10.1371/journal.pone.0126490

**Published:** 2015-04-27

**Authors:** Chunyan Zhu, Hongling Yang, Qingshan Geng, Qingling Ma, Yan Long, Cheng Zhou, Ming Chen

**Affiliations:** 1 Department of Preventive Medicine, School of Public Health, Guangzhou Medical University, Guangzhou, 510182, China; 2 Department of Laboratory, Guangzhou Women and Children’s Medical Centre, Guangzhou Medical University, Guangzhou, 510623, China; 3 Guangdong General Hospital, Guangdong Academy of Medical Science, Guangzhou, 510080, China; University of Catanzaro Magna Graecia, ITALY

## Abstract

**Objective:**

The relationship between gestational diabetes mellitus (GDM) and oxidative stress has not been fully elucidated. This study examined the association between biomarkers of oxidative stress and GDM.

**Methods:**

We conducted a case-control study which included 36 women presenting with GDM and 36 asymptomatic matched control subjects who visited Guangzhou Women and Children’s Medical Centre, China, from June 2012 to December 2012. Pregnant women were prospectively recruited to the study, and blood samples were collected at the time of a routine oral glucose tolerance test. These samples were then analyzed for levels of endocrine and surrogate markers of oxidative stress.

**Results:**

Compared to control subjects, women with GDM exhibited elevated values for plasma glucose, insulin, and insulin resistance (IR), and showed reduced HOMA pancreatic β-cell function (HOMA-B), insulin sensitivity index (ISI), insulinogenic index, and corrected insulin response at 24–28 weeks gestation. A bivariate logistic regression analysis showed that levels of high-sensitivity C reactive protein (hs-CRP) and high fluorescence reticulocytes at fasting, and hs-CRP in a 1-h OGTT, were significantly associated with GDM. A linear regression analysis showed that levels of hs-CRP (P = 0.003) and reticulocytes (P = 0.029) at fasting were associated with IR, and levels of hs-CRP (P = 0.002) and monocytes (P = 0.006) in a 1-h OGTT were associated with ISI.

**Conclusions:**

Pregnant women with GDM developed a pathological IR and exhibited β-cell dysfunction. Their decreased ability to compensate for oxidative stress was associated with increased IR and a reduced ISI, which might be important factors in GDM.

## Introduction

Gestational diabetes mellitus (GDM) is a common metabolic complication of pregnancy, and is associated with increased rates of perinatal complications. Additionally, studies have shown that both women diagnosed as GDM and their offspring are at an increased risk for developing diabetes mellitus in later life [1–2]. Pregnancy is a progressively hyperglycemic period of life, and is associated increasing insulin resistance starting at midgestation [3]. Women with GDM develop an increased severity of insulin resistance which can disrupt the intrauterine milieu, leading to abnormal fetal growth [4].

In GDM, glucose tolerance and metabolism as well as insulin resistance are altered, and the pathophysiologic mechanisms underlying these changes are not completely understood. However, all of these changes are accompanied by oxidative stress [5]. The two main pathological mechanisms known to induce GDM involve the biochemical pathways leading to insulin resistance and chronic subclinical inflammation [6].

Insulin resistance is characterized by the inability of tissues to respond to insulin, and pancreatic beta cells compensating for this inability by secreting increased amounts of insulin. GDM results when the increased insulin secretion cannot compensate for the pregnancy-induced insulin resistance [7]. Oxidative stress is the common factor which underlies insulin resistance. Inflammation is a well recognized manifestation of oxidative stress, and the various pathways that generate inflammatory mediators (e.g. adhesion molecules and interleukins) are all induced by oxidative stress [8]. It has been suggested that prolonged stimulation of acute and chronic inflammation may be involved in the pathogenesis of insulin resistance [9]. While the relationship between GDM and oxidative stress has not been fully elucidated, recent evidence suggests the involvement of oxidative stress biomarkers such as catalase and lipoperoxides [10]. Additionally, superoxide dismutase activity and protein carbonyl content in the placenta [11] also appear related to GDM. The levels of acute phase protein (AP protein) may reflect the levels of stress response. The levels of positive phase AP proteins such as C-reactive protein (CRP) and ceruloplasmin (CER) were increased during periods of stress response, while levels of the negative phase AP protein transferrin (TRF) were decreased.

The incidence of GDM in China is rising, and consequently, GDM has become an important health issue. However, few studies have been conducted which examined levels of oxidative stress and GDM-associated insulin resistance among pregnant women in China.

There is a clear need for additional studies on markers that can be used to identify and monitor GDM. The present study was conducted to examine the association of various biomarkers of oxidative stress with GDM among pregnant women in Guangzhou, China.

## Materials and Methods

### Study participants

The enrolled participants were recruited from a prospective open cohort of pregnant women followed in Guangzhou, China, who were attending the antenatal clinic at Guangzhou Women and Children’s Medical Centre (GWCMC). From June 2012 to December 2012, a total of 325 pregnant women were recruited to the study during their first antenatal visit by a clinical research nurse. Subjects with pre-existing diabetes, a hypertensive disorder complicating their pregnancy, pre-eclampsia or a history of multiple pregnancies were excluded from the study.

The protocol for this study was approved by the ethics review committees of Guangzhou Medical University, and all study participants provided their voluntary signed informed consent.

### Study design

This study was conducted utilizing a case-control design. All the participants were from the same cohort and followed up starting from their first antenatal care visit until delivery. All pregnant women attending GWCMC are routinely screened for GDM at ~ 24 and 28 weeks gestation using an oral glucose tolerance test (OGTT). Women who presented with GDM, (as determined by the OGTT) were included in the study. Among the 325 subjects recruited, 36 were diagnosed with GDM after overnight fasting followed by a 75 g glucose load at ~ 24 and 28 weeks gestation. GDM was diagnosed according to the American Diabetes Association diagnostic criteria published in the pregnancy guidelines in the Standards of Medical Care in Diabetes, 2011 [12].

An equal number of matched, normal (non-GDM) control subjects was randomly selected from the group of pregnant women. The normal control subjects were matched with GDM women for maternal age, gestational weeks, gravidity, parity, and BMI. According to the time sequence of registration, the control subjects were selected at random by the same nurse after delivery to ensure the controls did not have GDM, hypertensive disorders or pre-eclampsia during the gestation period. All women enrolled in this study delivered a live birth.

### Data collection

The height and weight of each subject measured while wearing light clothing and no shoes were used to calculate body mass index. Weight was measured to the nearest 0.1 kg using a digital electronic scale, and height was measured to the nearest 0.1 cm using a stadiometer. Body mass index (BMI) was determined at the first antenatal visit, and calculated as weight divided by height squared (kg /m^2^).

### Blood collection

Subjects at ~ 24 and 28 weeks gestation ate a high carbohydrate diet for 3 days, and then fasted overnight; after which glucose tolerance was assessed using a 75-g OGTT. Venous blood samples for routine pathological tests were collected immediately before and 1 hour after the 75 g glucose load. These samples were immediately centrifuged and stored at—80°C.

### Insulin resistance and insulin secretion assays

Levels of plasma glucose were measured using a biochemical analyzer (7600; High-Technologies Corporation, Japan), and plasma insulin levels were measured using an immunochemiluminescent insulin assay (Abbott Diagnostics). Insulin resistance (IR) and pancreatic cell function were evaluated by homeostasis model assessment (HOMA). Indices of insulin resistance included fasting serum insulin (INS_0_), 1-h serum insulin (INS_60_), and HOMA-IR. Insulin secretion indices included the insulin sensitivity index (ISI), insulinogenic index, the corrected insulin response (CIR) and HOMA pancreatic β-cell function (HOMA-B). HOMA-IR was calculated as fasting plasma glucose [(GLU_0_, mmol/L) x INS_0_ (mIU/L)] / 22.5]; HOMA-B was calculated as INS_0_ x 20 / (GLU_0_-3.5); HOMA-ISI was calculated as 1 / [1-h plasma glucose (GLU_60_) x INS_60_]; insulinogenic index was calculated as (INS_60_—INS_0_) / (GLU_60_—GLU_0_); CIR was calculated as INS_60_ / GLU_60_ x (GLU_60_-70).

### Stress index assays

Assays for serum levels of ceruloplasmin (CER), hs-CRP, and transferrin (TRF) were performed using enzyme-linked immunonephelometry (Beckman Coulter Diagnostics); 3-nitrotyrosine was identified using double-antibody sandwich ABC-ELISA (IBL International GmbH Diagnostics). Hematologic parameters were measured using an automated hematology analyzer (XE-2100; Sysmex, Kobe, Japan).

### Data analysis

All data were analyzed using SPSS for Windows, Version 13.0. Chicago, IL; SPSS Inc. Correlations between parameters were evaluated using Pearson’s or Spearman’s correlation coefficients; comparisons between groups of data were done using the Student’s t-test or Mann—Whitney U test. Conditional univariate and bivariate logistic regression analyses were used to determine the influence of significant independent variables on GDM outcomes. Associations between fasting HOMA-IR or ISI and stress factors were determined by estimating the natural logarithm (ln) of fasting HOMA-IR or 1-h ISI as a linear function of stress factors using regression. A two-sided P-value < 0.05 was considered statistically significant.

### Statistical power

With 36 cases plus one control subject for each case, a 5% significance level in a two-sided test, and mean insulin concentrations of 8.60μU/mL for GDM pregnant women and 6.53μU/mL (SD 3.1) for non-GDM pregnant women (according to our pilot study), the study had>80.0% power.

## Results

### Sociodemographic factors

The study cohort comprised a total of 72 subjects (36 GDM subjects and controls, respectively) with lengths of gestation ranging from 24 to 28 weeks (Table 1). Among the study participants, > 75% was aged 25 to 34 years (range 23–40 years), 52.8% were gravidity 1, and > 80% were parity 1. There were no significant differences in sociodemographic factors between the GDM and control groups at the time of recruitment.

**Table 1:** Sociodemographic characteristics of women in the GDM and control cohorts.

		Case (n = 36)	Control (n = 36)	P value
Age (year) *	Mean ± SD	31.17 ± 4.03	30.44 ± 3.70	0.572
Age group (year)	19–24	2 (5.6%)	3 (8.3%)	-
	25–34	27 (75.0%)	29 (80.6%)	
	≥ 35	7 (19.4%)	4 (11.1%)	
Gestational weeks when OGTT (week) *	Mean ± SD	26.06 ± 3.11	25.97 ± 2.73	0.931
Gravidity *	1	19 (52.8%)	19 (52.8%)	1.000
	≥ 2	17 (47.2%)	17 (47.2%)	
Parity *	1	29 (80.6%)	30 (83.3%)	0.616
	≥ 2	7 (19.4%)	6 (16.7%)	
Pre-pregnancy BMI (weight/height^2^) *	Mean ± SD	21.19±1.86	20.45±2.18	0.112
	18.5–23.9 (normal)	29 (80.6%)	22 (61.1%)	Reference
	< 18.5 (underweight)	4 (11.1%)	10 (27.8%)	0.197
	> 23.9 (overweight or obese)	3 (8.3%)	4 (11.1%)	0.641
Family history of diabetes	No	27 (75.0%)	33 (91.7%)	0.058
	Yes	9 (25.0%)	3 (8.3%)	
Family history of hypertension	No	28 (77.8%)	30 (83.3%)	0.551
	Yes	8 (22.2%)	6 (16.7%)	

* Maternal age, gravidity, parity, BMI and gestational weeks were determined at the time of recruitment. Control subjects were matched against subjects with GDM.

GDM, gestational diabetes mellitus; BMI, body mass index

### Insulin function indices

At fasting, values for plasma glucose, insulin, and HOMA-IR were higher, and values for HOMA-B were lower in the GDM group compared to those in the non-GDM group. In a 1-h OGTT, values for plasma glucose and insulin were higher, and values for HOMA-ISI, insulinogenic index and CIR were lower in the GDM group (Table 2). The values for ln(IR) and ln(INS_0_) in the entire cohort of 72 subjects were significantly correlated with fasting glucose concentrations (Pearson’s r = 0.983, P < 0.001; Pearson’s r = 0.549, P = 0.002, respectively). Results of a 1-h OGTT for the entire cohort showed significant correlations between ln(ISI) and ln(INS_60_) (Pearson’s r = 0.947, P < 0.001; ln(ISI) and ln(insulinogenic index) (Pearson’s r = 0.262, P < 0.05), and ln(ISI) and glucose concentrations(Pearson’s r = 0.947, P < 0.01).

**Table 2:** Insulin secretory capacity and sensitivity of women in the GDM and control cohorts at fasting and a 1-h OGTT.

	Fasting			1-h OGTT		
	Case	Control	P	Case	Control	P
Plasma glucose (mmol/L)	4.63 ± 0.55	4.20 ± 0.31	< 0.001	10.24 ± 1.33	7.57 ± 1.23	< 0.001
Insulin (μU/mL)	8.93 ± 3.80	6.80 ± 2.79	0.008	89.18 ± 34.98	70.73 ± 34.32	0.011
HOMA-IR	1.88 ± 1.02	1.28 ± 0.54	0.003	-	-	-
HOMA-B	173.76 ± 69.84	256.06 ± 224.76	0.038	-	-	-
HOMA-ISI	-	-	-	0.001 ± 0.001	0.003 ± 0.002	< 0.001
Insulinogenic index	-	-	-	14.97 ± 8.28	20.42 ± 10.23	0.006
CIR (×10^3^)	-	-	-	4.48 ± 2.34	8.73 ± 4.69	< 0.001

Data are means ± SD.

GDM, gestational diabetes mellitus; OGTT, oral glucose tolerance test; HOMA-IR, HOMA insulin resistance index; HOMA-B, HOMA pancreatic β-cell function; HOMA-ISI, HOMA insulin sensitivity index; CIR, corrected insulin response

### Stress factors

Comparisons of stress factor indices between the GDM and control groups at fasting and 1-h OGTT were done using the Student’s t test or Mann—Whitney U test (Table 3). Compared to control subjects, 24 and 28-week gestation women with GDM showed significantly higher concentrations of hs-CRP, NEUTs, RBCs, RETs and HFRs, but lower values of MCV compared to control subjects. In a 1-h OGTT, the GDM cohort also showed significantly higher concentrations of hs-CRP and RBCs, and lower concentrations of MONOs compared to the control group. Other variables showed no significant differences between cohorts at either fasting or a 1-h OGTT.

**Table 3:** Biological markers of stress factors for women in the GDM and control cohorts at fasting and a 1-h OGTT.

	Fasting			1-h OGTT		
	Case	Control	P	Case	Control	P
hs-CRP (mg/L)	4.46 ± 3.07	2.53 ± 1.66	0.001	4.42 ± 3.14	2.54 ± 1.65	0.004
CER (g/L)	0.64 ± 0.11	0.63 ± 0.10	0.739	0.72 ± 0.10**	0.70 ± 0.11**	0.560
3-NT (nmol/L)	83.38 ± 65.04	94.65 ± 65.13	0.386	119.72 ± 85.19**	134.68 ± 83.92**	0.353
TRF (g/L)	3.15 ± 0.41	3.15 ± 0.49	0.994	3.32 ± 0.55**	3.22 ± 0.45	0.449
RBC (10^12^/L)	3.84 ± 0.40	3.64 ± 0.30	0.023	3.77 ± 0.39**	3.60 ± 0.28*	0.037
HGB (g/dL)	113.31 ± 12.46	112.39 ± 10.45	0.736	111.11 ± 12.23**	110.92 ± 9.61**	0.940
HCT (%)	34.78 ± 3.33	34.49 ± 2.75	0.695	34.22 ± 3.14	34.13 ± 2.56	0.899
MCH (pg)	29.74 ± 3.57	30.88 ± 1.96	0.101	29.67 ± 3.62	30.83 ± 1.95	0.096
MCHC (g/dL)	325.36 ± 9.69	325.50 ± 7.98	0.947	324.22 ± 10.01	324.69 ± 8.26	0.828
MCV (fL)	91.23 ± 9.42	94.82 ± 5.05	0.049	91.34 ± 9.45	94.9 2± 5.11	0.051
RET (10^12^/L)	0.09 ± 0.02	0.08 ± 0.03	0.046	0.09 ± 0.02	0.08 ± 0.03	0.078
HFR (%)	1.64 ± 1.19	0.96 ± 0.91	0.008	1.36 ± 1.15*	0.93 ± 1.10	0.111
MFR (%)	12.37 ± 3.88	10.65 ± 4.55	0.090	11.42 ± 3.81**	9.67 ± 4.46**	0.078
LFR (%)	85.99 ± 4.96	88.39 ± 5.41	0.054	87.22 ± 4.75**	89.40 ± 5.47**	0.075
WBC (10^9^/L)	11.13 ± 2.46	10.03 ± 2.30	0.054	10.75 ± 2.37**	9.78 ± 2.10*	0.070
MONO (10^9^/L)	0.53 ± 0.14	0.53 ± 0.15	1.000	0.38 ± 0.12**	0.44 ± 0.12**	0.041
NEUT (10^9^/L)	8.47 ± 2.10	7.44 ± 1.93	0.034	8.52 ± 2.10	7.63 ± 1.83*	0.057
LYMPH(10^9^/L)	2.00 ± 0.57	1.93 ± 0.53	0.570	1.73 ± 0.53**	1.61 ± 0.43**	0.282
PLT (10^9^/L)	253.36 ± 60.03	239.72 ± 56.59	0.325	250.72 ± 57.95	236.86 ± 56.25	0.307
MPV (%)	10.76 ± 2.02	11.07 ± 0.90	0.406	10.75 ± 2.01	10.99 ± 0.89*	0.526
PDW (fL)	13.12 ± 2.95	13.35 ± 1.87	0.686	13.09 ± 2.88	13.25 ± 2.11	0.794
PCT (%)	0.25 ± 0.07	0.25 ± 0.05	0.745	0.25 ± 0.07	0.24 ± 0.05	0.638

Data are means ± SD.

* Comparison of fasting and 1-h OGTT values of case and control groups, respectively, P < 0.05.

** Comparison of fasting and 1-h OGTT values of case and control groups, respectively, P < 0.01.

GDM, gestational diabetes mellitus; OGTT, oral glucose tolerance test; hs-CRP, high-sensitivity C reactive protein; CER, ceruloplasmin; NT, nitrotyrosine; TRF, transferrin; RBC, red blood cell; HGB, hemoglobin; HCT, hematocrit; MCH, mean cell hemoglobin; MCHC, mean corpuscular hemoglobin concentration; MCV, mean cell volume; RET, reticulocyte; HFR, high fluorescence reticulocytes; MFR, middle fluorescence reticulocytes; LFR, low fluorescence reticulocytes; WBC, white blood cell; MONO, monocyte; NEUT, neutrophil; LYMPH, lymphocyte; PLT, platelets; MPV, mean platelet volume; PDW, red blood cells volume distribution width; PCT, plateletcrit

### Correlations with indices of insulin function

We next examined the correlations between indices of fasting and 1-h insulin function at 24 to 28 weeks gestation and various stress factors (Table 4). The results showed that pooled (GDM group plus control group) fasting HOMA-IR and INS indices were significantly correlated with levels of hs-CRP (Spearman's rho = 0.441 and 0.437, respectively). Additionally, fasting HOMA-IR and INS indices were significantly correlated with the numbers of RETs (0.370 and 0.363, respectively), and HFRs (0.253 and 0.251, respectively). Pooled 1-hour INS results were significantly correlated with 1-h hs-CRP levels (0.275), and 1-h ISI values were negatively correlated with 1-h hs-CRP levels (-0.345). Accordingly, 1-h ISI values were positively correlated with the numbers of MONOs (0.266) and LFRs (0.258), but negatively correlated with the numbers of RBCs (-0.238), RETs (-0.333), HFRs (-0.239), and MFRs (-0.255). Finally, 1-h INS levels were positively correlated with RET numbers (0.261) and negatively correlated with MONO numbers (-0.237). No correlations were evident between fasting HOMA-B results or 1-h insulinogenic index and the respective stress factors.

**Table 4:** Correlation between high sugar stress factors and HOMA-IR or HOMA-ISI in women diagnosed as GDM and controls at fasting and a 1-h OGTT.

	Fasting		1-h OGTT	
	HOMA-IR	Insulin	HOMA-ISI	Insulin
hs-CRP (mg/L)	0.441**	0.437**	-0.345**	0.275*
CER (g/L)	-0.094	-0.095	0.089	-0.040
3-NT (nmol/L)	0.078	0.070	0.021	-0.023
TRF (g/L)	-0.034	-0.031	-0.131	0.194
RBC (10^12^/L)	0.139	0.138	-0.238*	0.183
HGB (g/dL)	0.122	0.123	0.071	0.041
HCT (%)	0.117	0.113	0.135	0.107
MCH (pg)	-0.114	-0.106	-0.187	-0.190
MCHC (g/dL)	0.080	0.091	-0.136	-0.166
MCV (fL)	-0.129	-0.121	-0.106	-0.071
RET (10^12^/L)	0.370**	0.363**	-0.333**	0.261*
HFR (%)	0.253*	0.251*	-0.239*	0.198
MFR (%)	0.191	0.187	-0.255*	0.218
LFR (%)	-0.204	-0.200	0.258*	-0.218
WBC (10^9^/L)	0.168	0.151	-0.113	0.074
MONO (10^9^/L)	0.057	0.061	0.266*	-0.237*
NEUT (10^9^/L)	0.161	0.138	-0.129	0.083
LYMPH (10^9^/L)	0.141	0.157	0.156	0.161
PLT (10^9^/L)	0.082	0.079	-0.039	0.052
MPV (%)	-0.031	-0.045	0.066	0.039
PDW (%)	0.050	0.032	0.090	0.071
PCT (%)	0.008	-0.001	-0.032	-0.014

Data are Spearman's rho.

* P < 0.05

** P < 0.01

HOMA-IR, HOMA insulin resistance index; HOMA-ISI, HOMA insulin sensitivity index; GDM, gestational diabetes mellitus; OGTT, oral glucose tolerance test; hs-CRP, high-sensitivity C reactive protein; CER, ceruloplasmin; NT, nitrotyrosine; TRF, transferrin; RBC, red blood cell; HGB, hemoglobin; HCT, hematocrit; MCH, mean cell hemoglobin; MCHC, mean corpuscular hemoglobin concentration; MCV, mean cell volume; RET, reticulocyte; HFR, high fluorescence reticulocytes; MFR, middle fluorescence reticulocytes; LFR, low fluorescence reticulocytes; WBC, white blood cell; MONO, monocyte; NEUT, neutrophil; LYMPH, lymphocyte; PLT, platelets; MPV, mean platelet volume; PDW, red blood cells volume distribution width; PCT, plateletcrit

### Conditional logistic regression analysis

Using a sample size of 72, we performed a multivariable conditional logistic regression analysis to assess the association of GDM with the putative predictive biomarkers which had previously shown an association with GDM at levels of P ≤ 0.05 in the univariate analysis (Table 3). Of these, six stress factors assayed at fasting (hs-CRP levels; RBC, NEUT, RET, MCV, and HFR numbers) and three stress factors in the 1-h OGTT (hs-CRP levels; RBC and MONO numbers) showed significant (P ≤ 0.05) associations with GDM outcome. A multivariable bivariate conditional logistic regression analysis was then performed to assess the association of the stress factors at fasting and the 1-h OGTT results, respectively, with GDM outcome when adjusted for all possible pairings. The details of this analysis are presented in Table 5. At fasting, the following variables were significantly associated with GDM outcome: hs-CRP (OR: 1.41; 95% CI: 1.02–1.96, P = 0.040) and HFR (OR: 2.05; CI: 1.00–4.21, P = 0.005). Results from analysis of the 1-h OGTT showed that hs-CRP (OR: 1.52; CI: 1.07–2.16, P = 0.021) was the only variable significantly (P ≤ 0.05) associated with outcome.

**Table 5:** Multivariable conditional logistic regression analysis of explanatory variables at fasting and a 1-h OGTT against outcome of GDM.

	Fasting		1-h after OGTT	
	Odds ratio (95% CI)	P value	Odds ratio (95% CI)	P value
Hs-CRP	1.41 (1.02, 1.96)	0.040	1.52 (1.07, 2.16)	0.021
HFR	2.05 (1.00, 4.21)	0.050	-	-
MCV	0.99 (0.85, 1.14)	0.851		
MONO	-	-	0.002 (0.001, 1.113)	0.054
NEUT	1.33 (0.84, 2.09)	0.225	-	-
RBC	6.73 (0.76, 59.98)	0.088	5.62 (0.71, 44.45)	0.102
RET	1.64 (0.59, 4.61)	0.347	-	-

GDM, gestational diabetes mellitus; OGTT, oral glucose tolerance test; hs-CRP, high-sensitivity C reactive protein; HFR, high fluorescence reticulocytes; MCV, mean cell volume; MONO, monocyte; NEUT, neutrophil; RBC, red blood cell; RET, reticulocyte

### Linear regression analysis

A multivariable linear regression analysis was also performed to assess the associations of HOMA-IR and ISI data with the stress factors which were significantly correlated with dependent variables in the Spearman’s correlation analysis (Table 4). Given the correlations among HFR, MFR, and LFR, only HFR was included in the multivariable linear regression used to assess the association of IR or ISI data with the stress factors when adjusted for all possible pairings. The details of this analysis are presented in Table 6. At fasting, the following variables were associated with HOMA-IR results: hs-CRP levels (coefficient = 0.06, P = 0.003) and RET numbers (4.77, P = 0.029). The variables associated with ISI values in the 1-h OGTT were hs-CRP levels (0.08, P = 0.002) and MONO numbers (-0.31, P = 0.006).

**Table 6:** Multivariable linear regression analysis of explanatory variables against HOMA-IR or HOMA-ISI, at fasting and a 1-h OGTT.

	Ln(IR) at fasting^a^			Ln(ISI) at 1-hr OGTT^b^		
	B ^c^ (95% CI)	Beta ^d^	P	B ^c^ (95% CI)	Beta ^d^	P
Constant	-0.29 (-0.68, 0.08)	-	0.122	6.76 (6.32, 7.21)	-	< 0.001
Hs-CRP	0.06 (0.02, 0.11)	0.34	0.003	0.08 (0.03, 0.13)	0.35	0.002
HFR	0.02 (-0.09, 0.13)	0.05	0.702	0.06 (-0.06, 0.17)	0.11	0.333
MONO	-	-	-	-1.43 (-2.43, -0.42)	-0.31	0.006
RET	4.77 (0.51, 9.04)	0.24	0.029	3.31 (-1.90, 8.52)	0.14	0.209
RBC	-	-	-	0.30 (-0.06,0.66)	0.18	0.104

^a^: The independent variables included hs-CRP, HFR, and RET.

^b^: The independent variables included hs-CRP, HFR, MONO, RET, and RBC.

^c^: Non-standardized coefficients.

^d^: Standardized coefficients.

HOMA-IR, HOMA insulin resistance index; HOMA-ISI, HOMA insulin sensitivity index; OGTT, oral glucose tolerance test; hs-CRP, high-sensitivity C reactive protein; HFR, high fluorescence reticulocytes; MONO, monocyte; RET, reticulocyte; RBC, red blood cell

## Discussion

The present study primarily examined the association of oxidative stress indicators with GDM in pregnant women. The results indicated that pregnant women with GDM developed a pathological increase in IR and displayed β-cell dysfunction. The decreased ability to compensate for oxidative stress found among these women was associated with increased IR and a reduced ISI, which might be important factors in GDM.

Previous studies have shown that GDM is associated with insulin resistance and β-cell dysfunction [12–13]. In our study, this association was demonstrated by increased plasma glucose and insulin concentrations in women with GDM both at fasting and a 1-h OGTT, when compared to the corresponding concentrations in control subjects. We also found that HOMA-IR values were increased while HOMA-B values were decreased in GDM subjects. Additionally, the differences in HOMA-ISI, insulinogenic index, and CIR values between GDM and matched control subjects were highly significant. The current study confirms that GDM is associated with increased insulin resistance and β-cell dysfunction, as well as reduced insulin sensitivity and secretion.

It is well accepted that inflammatory and stress responses mediate insulin resistance [14], and inflammatory mediators play an important role in the development and progression of GDM. CRP, a classic acute-phase reactant, is a sensitive marker of inflammation in numerous pathologic conditions, and elevated CRP levels have been associated with abnormal metabolic conditions such as insulin resistance, hyperglycemia, and T2DM [15]. During pregnancy, increased CRP levels are associated with insulin resistance, maternal dysglycemia, and GDM [16, 17]. Additionally, reactive oxygen species (ROS) induce production of inflammatory mediators such as CRP, and thus play a causative role in these inflammatory processes [18]. Inflammatory proteins appear to gradually impair beta cell function and increase insulin resistance, which results in ineffective control of plasma glucose levels, and subsequent dysglycemia [6]. The present study showed that plasma hs-CRP levels were higher in pregnant women with GDM compared a cohort of control subjects both at fasting and a 1-h OGTT. These higher hs-CRP levels were manifested as an increased lipid peroxidation capacity and decreased antioxidant defense capacity of the glutathione system.

Diabetes is associated with increased oxidative stress as measured by lipid peroxidation and protein oxidation/nitration [19]. CER and Trf are acute-phase reactants and extracellular antioxidants. Trf circulates in the blood as a carrier protein for iron (two iron atoms per molecule). CER catalyzes the incorporation of iron into Trf without formation of toxic iron products; therefore, elevated plasma CER levels may signal abnormally high levels of oxidant stress [20]. Memişoğullari et al reported that serum CER and CRP levels were significantly higher, while Trf levels were significantly lower in a cohort of diabetic patients, compared to levels in control subjects [21]. NT is a product of tyrosine nitration mediated by reactive nitrogen species. The inflammation indicator 3-NT is also known to be an important surrogate marker of oxidative stress and protein nitration damage. Although circulating levels of CER, Trf, and 3-NT increased after the OGTT in our study, no significant differences were found between the GDM and control groups. Meanwhile, 3-NT levels were lower in GDM subjects compared to control subjects both at fasting and in the 1-h OGTT; however, the difference between the two groups was not statistically significant. This finding may have resulted from a relatively acute pregnancy-induced increase in antioxidant activity, due to increased superoxide dismutase levels. In the longer term, as overt diabetes becomes a chronic condition, oxidative stress is compounded by the formation of advanced anti-oxidative products which cause tissue damage [22].

A chronic low-grade activation of the immune system may contribute to the pathogenesis of type 2 diabetes. Accordingly, an increased pro-inflammatory state enhances WBC and endothelial cell activation, thereby promoting platelet aggregation and thrombus formation [23]. Elevated WBC counts may be indicative of a clinical or subclinical inflammation. An earlier study of 352 Pima Indians showed that high a WBC count was associated with reduced insulin sensitivity, and predictive for developing type 2 diabetes [24]. A study of South-East Asian women showed that women with an increased WBC count in early pregnancy had a significantly higher rate of GDM compared to women with a normal WBC count [25]. In the present study, women with GDM showed significantly increased neutrophil and WBC counts both at fasting and a 1-h OGTT. In contrast, monocyte counts were significantly lower among women with GDM compared to controls, and were independently associated with insulin sensitivity. Our results suggest that pregnant women with GDM have a decreased ability to compensate for oxidative stress, and that a prooxidant/oxidant imbalance is involved in increased stress-induced tissue damage that can result in metabolic disturbance and immune system malfunction.

Red blood cells possess a potent antioxidant protection mechanism which involves enzymatic and nonenzymatic pathways that transform ROS into less reactive intermediates [26]. Additionally, RBC alterations have been linked to increased oxidative states and levels of inflammatory markers. A study of 60 pregnant women (30 with GDM and 30 w/o GDM) in Kosovo showed that women with GDM had higher RBC counts compared to those in a control group [27], and a similar conclusion was reported in another study conducted among pregnant women in China [28]. In our current study, significantly increased RBC counts were observed among women with GDM both at fasting and a 1-h OGTT compared to counts in the control group. Additionally, increased RET counts and HFR percentages, but lower MCV values were observed at fasting among women with GDM. Our results suggest that altered blood cell physiology may have been a consequence of chronic long-term increased oxidative stress.

A limitation of our present study is that the data were collected at ~ 24 and 28 weeks gestation. Methods for prevention of GDM may have been more effective if employed prior to this unstable period of pregnancy, and further research is needed to identify women at this stage of disease development. Another limitation is the small number of patients, which may have affected the study results. However, despite the relatively small sample sizes, the validity of our results are strengthened by the close matching of control subjects against GDM subjects. Women were matched for age, gestational weeks, gravidity, parity and BMI, thus negating these parameters as confounders.

In conclusion, our results show that pregnant women who undergo a 1-h OGTT demonstrate hematological alterations, disturbances in their oxidant—antioxidant balance, and increased levels of inflammatory factors. The human body compensates for stress damage by inducing a systemic nonspecific reaction to prevent diseases characterized by increased levels of acute-phase proteins (e.g. hs-CRP and CER), protein degradation products (such as 3-NT), and decreased levels of stress-related cells (such as WBCs, RBCs, and MONOs). Our findings suggest that the decreased ability of pregnant women with GDM to compensate for oxidative stress was manifested as increased insulin resistance, reduced insulin sensitivity, and β-cell dysfunction, all of which may play important roles in GDM.
